# Long-term safety and efficacy of renal sympathetic denervation in comparison to a population-based cohort: a propensity-matching approach

**DOI:** 10.1097/HJH.0000000000004117

**Published:** 2025-08-11

**Authors:** Victor J.M. Zeijen, Martijn J. Tilly, Kari A. Saville, Bruno H.C. Stricker, Isabella Kardys, M. Kamran Ikram, Maryam Kavousi, Joost Daemen

**Affiliations:** a Department of Cardiology, Thorax Center; b Department of Epidemiology; c Department of Neurology, Erasmus University Medical Center, Rotterdam, the Netherlands

**Keywords:** blood pressure, cohort studies, glomerular filtration rate, hypertension, kidney, sympathectomy

## Abstract

**Objective:**

To evaluate the long-term changes in risk of cardiovascular outcomes and blood pressure (BP) in hypertensive patients treated with renal sympathetic denervation (RDN) as compared to hypertensive controls from a population-based cohort.

**Methods:**

This prospective cohort study included patients with office systolic blood pressure (SBP) at least 140 mmHg and/or diastolic BP at least 90 mmHg. Patients treated with RDN were matched to hypertensive controls from the population-based Rotterdam Study using one-to-many variable-ratio propensity score matching. The primary safety outcome was a composite endpoint of myocardial infarction, coronary revascularization, stroke, renal failure and mortality. The primary efficacy outcome was the 5-year change in office SBP.

**Results:**

A total of 53 RDN patients were matched to 238 population-based controls. Median age [25th–75th percentile] was 60.5 [56.5–68.4] years (46% female). Baseline BP ±SD was 166.1/95.5 ± 20.6/10.9 mmHg. Patients were prescribed 2.8 [1.5–4.5] defined daily dosages of antihypertensive drugs. The incidence of the primary safety outcome was similar among the RDN group and the control group at 5 years [13 vs. 18%; hazard ratio 0.93; 95% confidence interval (CI) 0.36–2.38; *P* = 0.87]. The 5-year change in SBP was −12.0 [−18.0, −6.0] mmHg in the RDN group (*P* < 0.001) and −14.9 [−22.5 to −7.3] mmHg in the control group (*P* < 0.001), with no significant between-group difference [2.9 (−6.6 to 12.4) mmHg; *P* = 0.55].

**Conclusion:**

Patients with uncontrolled hypertension undergoing RDN did not have a significantly lower risk for future adverse cardiovascular events as compared to hypertensive controls from a population-based study. No difference in office BP was observed at 5 years. While real-world observational data could provide valuable insights, randomized trials are needed to confirm the role of RDN in improving long-term outcomes.

## INTRODUCTION

Hypertension remains an important cardiovascular risk factor, accounting for a reduction in cardiovascular disease-free life-years of 3–5 years [1,2]. Reducing systolic blood pressure (SBP) by 5–10 mmHg has demonstrated to lower the cardiovascular risk by 10–20%, respectively [3,4]. Yet, blood pressure (BP) remains uncontrolled in up to 50% of all patients with an established diagnosis of hypertension [5,6].

During the last decade, the safety and efficacy of renal sympathetic denervation (RDN) in patients with uncontrolled hypertension (both on and off medications) has been studied in six randomized sham-controlled trials. At 2–6 months, patients treated with RDN experienced reductions of 5–10 mmHg in ambulatory SBP, as compared to control patients who either had smaller reductions of 1–7 mmHg or were prescribed more antihypertensive drugs [7–12]. At 3 years, the longest follow-up data from two randomized trials revealed reductions in ambulatory SBP of 15–19 mmHg following RDN, which were substantially larger than the reductions in sham-control patients of 0–9 mmHg, while on a similar antihypertensive drug regimen [13,14]. Up until 10 years following RDN, registry studies showed sustained reductions in ambulatory SBP ranging between 8 and 21 mmHg, in the absence of intensification of the antihypertensive drug regimen [15–19]. Changes in renal function (estimated glomerular filtration rate; eGFR) of −1 to −2 ml/min/1.73 m^2^ per year have been observed up to 10 years following RDN, which are in line with the natural course of renal function in hypertensive patients [15–20].

To date, no comparative data beyond 36 months are available on RDN as compared to a control group. As of to date, randomized studies comparing long-term cardiovascular outcomes between RDN patients and control patients are lacking, and currently available long-term data are limited to single-arm clinical registries. These limitations could be partly overcome by using a population-based cohort study for the sampling of control patients. For this purpose, we took advantage of the Rotterdam Study, an ongoing population-based study which has run for over 30 years and includes almost 20 000 community-dwelling participants [21]. The aim of the current study was to evaluate the effect of RDN in patients with hypertension on cardiovascular risk, renal function, BP and antihypertensive drug regimen at 5 years, as compared to a control group of patients with hypertension who were drawn from a population-based cohort.

## METHODS

### Study design

The current prospective cohort study was a joint effort between the departments of Cardiology and Epidemiology of the Erasmus University Medical Center (Rotterdam, the Netherlands). Patients in the RDN group were sampled from the Rotterdam Renal Denervation Registry, which is a prospective clinical registry study of all patients treated with RDN within the Erasmus University Medical Center (Rotterdam, the Netherlands) [16]. Patients in the control group were sampled from the Rotterdam study, which is an ongoing prospective population-based cohort study in the city of Rotterdam (the Netherlands) (Supplemental Methods) [21].

### Study population

Within both the RDN group and the control group, adult patients with a documented history of hypertension who had an office SBP at least 140 mmHg and/or office diastolic blood pressure (DBP) at least 90 mmHg at baseline were included. Exclusion criteria were renal function (eGFR) less than 30 ml/min/1.73 m^2^ and renal artery anatomy ineligible for RDN (RDN group only).

### Study visits

Patients from both groups underwent a baseline visit, consisting of office BP measurements, renal function testing and data collection on medical history and antihypertensive drug regimen. After 5 years, patients were invited to a repeat visit, during which data was collected on office BP, antihypertensive drug regimen and renal function. The occurrence of cardiovascular or renal serious adverse events was continuously monitored using a protocolized annual outpatient clinic visit schedule (RDN group), or through a dedicated linkage to the general practitioner patient files and hospital registries (control group) [21]. Yearly ambulatory BP monitoring and visits to a dedicated hypertension clinic were routinely performed in the RDN group but not in the control group.

### Renal denervation procedure

Within the RDN group, the baseline visit was followed by noninvasive renal artery imaging (computed tomography angiography or magnetic resonance angiography) to confirm anatomical eligibility for the procedure. Afterwards, patients underwent RDN under local anesthesia or conscious sedation according to device-specific instructions for use. Patients were treated with a multitude of RDN devices, including the radiofrequency-based Covidien Oneshot, St. Jude EnligHTN, single-electrode Symplicity Flex, multielectrode Symplicity Spyral and Vessix devices and the ultrasound-based Paradise system. Following the procedure, patients remained hospitalized for 24 h and were afterwards discharged with 1 month of aspirin. To confirm renal artery patency, noninvasive renal artery imaging was repeated at 6 and/or 12 months following the index procedure.

### Definitions

Office BP was based on the average of two measurements. Prescribed antihypertensive drug regimen was displayed as the total number of defined daily dosages (DDD), the total number of drug classes and the use of individual drug classes (Supplemental Methods).

### Outcomes

The primary safety outcome was a composite of myocardial infarction, coronary revascularization, ischemic or hemorrhagic stroke, renal failure (defined as eGFR ≤15 ml/min/1.73 m^2^ or requirement for dialysis) and all-cause mortality (whichever occurred first) between baseline and 5-year follow-up. Secondary safety outcomes were the occurrence of the individual components of the primary safety outcome and cardiovascular mortality, noncardiovascular mortality and the change in renal function (eGFR) between baseline and 5-year follow-up.

The primary efficacy outcome was the change in office SBP (in mmHg) between baseline and 5 years. Secondary efficacy outcomes were the change in in office DBP (in mmHg) between baseline and 5 years, the percentage of patients with controlled BP at 5 years and the changes in the total number of antihypertensive drug DDDs and the number of antihypertensive drug classes between baseline and 5 years.

### Statistical analysis

Continuous variables were reported as mean ± standard deviation (SD) or median [25th–75th percentile] for normally and nonnormally distributed variables, respectively. Normality was assessed using histograms and quantile–quantile plots. Categorical variables were reported as number of patients (percentage).

To account for between-group differences in baseline characteristics, one-to-many variable-ratio nearest neighbor propensity score matching without replacement was performed. The caliper was set to 0.2 times the SD of the logit of the propensity score [22]. The preferred matching ratio was five matched controls per RDN patient (maximum 10). Propensity scores were calculated using a logistic regression model including the following baseline characteristics: age, biological sex, history of any cardiovascular disease, office SBP and DBP and the number of antihypertensive drug DDDs and classes. Propensity matching was performed for safety outcomes (within the complete study population) and for efficacy outcomes (subgroup of patients who completed their 5-year follow-up visit).

Time-to-event outcomes were analyzed using Cox proportional hazards models with the outcome variable as the dependent variable and treatment group (RDN group vs. control group) as the independent variable. Marginal hazard ratios were calculated and cluster-robust standard errors were implemented to adjust for data clustering within matched pairs. Matching weights were applied in all models to adjust for variable ratio matching. Outcomes were reported as the weighted cumulative incidence (number of events, percentages) in both treatment groups, the hazard ratio and the corresponding robust 95% confidence interval (CI) and the *P*-value from the robust score test. In case the outcome variable was not observed in one of the treatment groups, hazard ratios could not be reliably estimated and only robust score test *P* values were provided.

Continuous and categorical outcomes were analyzed using linear or logistic regression models, respectively, with the outcome as the dependent variable and treatment group as the independent variable. Estimates of the between-group difference (continuous outcomes) or odds ratio (categorical outcomes) were obtained using g-computation, while cluster-robust standard errors were implemented to adjust for data clustering within matched pairs [23]. Matching weights were applied in all models to adjust for variable ratio matching. Continuous outcomes within each of the treatment groups were reported as the weighted variable estimates for both groups, the between-group difference and the corresponding 95% CI (based on the cluster-robust standard error) and *P* value. Categorical outcomes were reported as weighted proportions for both groups, including the corresponding *P* value. With respect to reporting and statistical testing for baseline characteristics in matched samples, a similar approach was used as for the continuous and categorical outcomes.

For the purpose of sensitivity analyses, all safety and efficacy outcome analyses were repeated in the source population (consisting of both matched and nonmatched patients) to provide more insights in data patterns observed. Statistical analyses were performed in a similar way as in the matched sample; however, no formal between-group statistical testing was performed, and no weighting was applied.

Unless stated otherwise, two-tailed *P* values less than 0.05 were considered statistically significant. Statistical analyses were performed using R version 4.3.0 with the packages ‘MatchIt’, ‘marginaleffects’, ‘sandwich’, ‘survival’, ‘Hmisc’, ‘spatstat”and ‘ggplot2’ [24].

### Sample size calculation

Based on the available data, this study was statistically powered for the primary efficacy outcome (i.e. the change in SBP between baseline and 5 years), but not for the primary safety outcome. The sample size calculation was performed using a two-tailed paired sample *t* test to account for correlation within matched pairs. Within the RDN group, a change in office SBP of −18 ± 30 mmHg was anticipated based on previous long-term registry data [15,16]. Within the control group, SBP was assumed to remain stable over time, as no previous data were available on the temporal evolution of BP in hypertensive patients. Combined with an alpha-level of 0.05 and a power level of 0.80, a total sample size for the RDN group of at least 24 patients was required. The one-to-many variable-ratio matching allowed for a larger number of controls, thereby further increasing statistical power.

## RESULTS

### Study population

**FIGURE 1:**
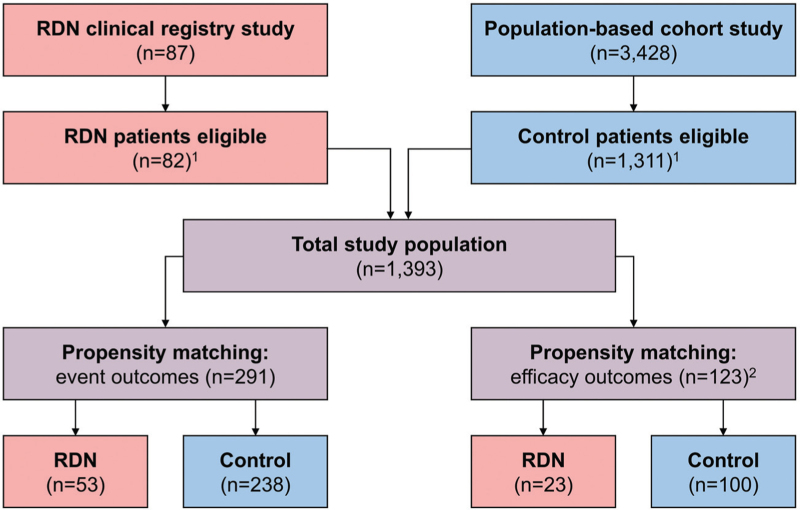
Study flowchart. ^1^A total of 2122 patients (5 RDN group, 2117 control group) were not eligible for participation in the current study (details provided in Supplementary Table 3). ^2^A total of 817 patients (37 RDN group, 780 control group) who did not complete the 5-year follow-up visit were excluded from the efficacy outcome analyses in the propensity-matched cohort. RDN, renal sympathetic denervation.

Between February 2006 and March 2022, a total of 1393 patients (82 RDN, 1311 control) were included in this study. Out of these, 53 RDN patients were propensity matched to 238 controls for clinical event outcomes. Five-year efficacy outcome data were available in 45 (55%) RDN patients and 531 (41%) controls, resulting in 23 RDN patients propensity matched to 100 control patients for efficacy outcomes (Fig. 1).

Overall, baseline characteristics were equally distributed among RDN patients and controls. Within the cohort matched for clinical event outcomes, median age at baseline was 60.5 [56.5–68.4] years and 134 (46%) patients were female. Median eGFR was 75.1 [63.2–87.2] ml/min/1.73 m^2^, and 89 (31%) patients had a history of cardiovascular disease. Baseline BP was 166.1/95.5 ± 20.6/10.9 mmHg, while patients were prescribed a median of 2.8 [1.5–4.5] DDDs of antihypertensive drugs. Some between-group differences were observed for specific antihypertensive drug classes (Table 1). Similar findings were observed within the cohort matched for efficacy outcomes (Supplemental Table 1).

**TABLE 1:** Baseline characteristics in the cohort matched for clinical event outcomes

	RDN group (*n* = 53)	Control group (*n* = 238)	*P* value^a^
Age (years), median [25th–75th percentile]	63.0 [55.4–70.7]	60.3 [56.7–67.5]	0.84
Female sex [*n* (%)]	27 (51)	107 (45)	0.42
BMI (kg/m^2^), median [25th–75th percentile]	28.5 [25.2–32.5]	29.8 [27.1–33.5]	0.26
Estimated glomerular filtration rate (ml/min/1.73 m^2^), median [25th–75th percentile]	72.5 [62.3–84.9]	75.6 [64.1–88.9]	0.41
Cardiovascular risk factors			
Hypertension [*n* (%)]	53 (100)	238 (100)	1.00
Dyslipidemia [*n* (%)]	50 (94)	215 (90)	0.41
Current smoking [*n* (%)]	10 (19)	49 (21)	<0.001
Former smoking [*n* (%)]	9 (17)	115 (48)	
Diabetes mellitus [*n* (%)]	9 (17)	78 (33)	0.048
History of cardiovascular disease			
Any cardiovascular disease [*n* (%)]	17 (32)	72 (30)	0.84
Myocardial infarction [*n* (%)]	2 (4)	14 (6)	0.62
Coronary revascularization [*n* (%)]	9 (17)	19 (8)	0.11
Stroke [*n* (%)]	6 (11)	22 (9)	0.71
Atrial fibrillation [*n* (%)]	2 (4)	37 (15)	0.06
Office blood pressure			
SBP (mmHg) (mean ± SD)	167.8 ± 18.3	165.7 ± 21.1	0.51
DBP (mmHg) (mean ± SD)	94.7 ± 13.9	95.6 ± 10.1	0.67
Antihypertensive drug regimen – summary measures			
Number of defined daily dosages, median [25th–75th percentile]	2.9 [1.5–4.8]	2.8 [1.5–4.4]	0.78
Number of classes, median [25th–75th percentile]	2.5 [1.0–2.9]	2.5 [1.2–3.0]	0.74
Antihypertensive drug regimen – drug classes			
Thiazide diuretics (*n* (%)]	34 (64)	121 (51)	0.07
Calcium channel blockers (*n* (%)]	27 (51)	71 (30)	0.009
Angiotensin-converting enzyme inhibitors [*n* (%)	9 (17)	92 (39)	0.003
Angiotensin receptor blockers [*n* (%)]	25 (47)	82 (35)	0.11
Mineralocorticoid receptor antagonist [*n* (%)]	1 (2)	46 (19)	0.009
Alpha blockers [*n* (%)]	10 (19)	7 (3)	0.005
Beta blockers [*n* (%)]	22 (42)	157 (66)	0.001
Direct renin inhibitor [*n* (%)]	0 (0)	0 (0)	1.00
Loop diuretics [*n* (%)]	2 (4)	20 (8)	0.42

RDN, renal sympathetic denervation; SD, standard deviation.

a Adjusted for data clustering (within matched pairs) and matching weights (due to variable ratio matching).

Baseline characteristics were distributed evenly among patients matched for clinical event outcomes (RDN *n* = 53; control *n* = 238) and patients matched for efficacy outcomes (RDN *n* = 23; control *n* = 100), except for the prevalence of previous cardiovascular disease, which was numerically higher among the cohort matched for clinical event outcomes (32%) as compared to cohort matched for efficacy outcomes (17%) among patients treated with RDN (Table 1, Supplemental Table 1). For the source population, baseline characteristics are displayed in Supplemental Table 2.

### Renal sympathetic denervation procedural characteristics

Among the 53 RDN patients that were propensity-matched to controls for clinical event outcomes, ultrasound RDN was performed in 19 (36%) patients, whereas radiofrequency RDN was performed in 34 (64%) patients. The median procedural duration was 59.5 [50.5–68.9] minutes while a median contrast volume of 75.0 [56.3–104.2] ml was used (Table 2).

**TABLE 2:** Renal denervation procedural characteristics in the cohort matched for efficacy outcomes

	RDN group (*n* = 53)
Procedural duration (min), median [25th–75th percentile]	59.5 [50.5–68.9]
Contrast volume (ml), median [25th–75th percentile]	75.0 [56.3–104.2]
RDN Device modality	
Paradise (US) [*n* (%)]	19 (36)
Symplicity Spyral (RF) [*n* (%)]	17 (32)
Symplicity Flex (RF) [*n* (%)]	4 (8)
EnligHTN (RF) [*n* (%)]	12 (23)
Vessix (RF) [*n* (%)]	1 (2)
OneShot (RF) [*n* (%)]	0 (0)
Number of bilateral emissions	
Paradise (US), median [25th–75th percentile]	5.5 [3.9–5.8]
Symplicity Spyral (RF), median [25th–75th percentile]	21 [17.3–23.9]
Symplicity Flex (RF), median [25th–75th percentile]	10.5 [10.0–11.0]
EnligHTN (RF), median [25th–75th percentile]	15.5 [15.0–18.0]
Vessix (RF), median [25th–75th percentile]	4.0 [4.0–4.0]
OneShot (RF), median [25th–75th percentile]	N/A

RDN, renal sympathetic denervation; RF, radiofrequency; SD, standard deviation; US, ultrasound.

### Safety outcomes

**FIGURE 2:**
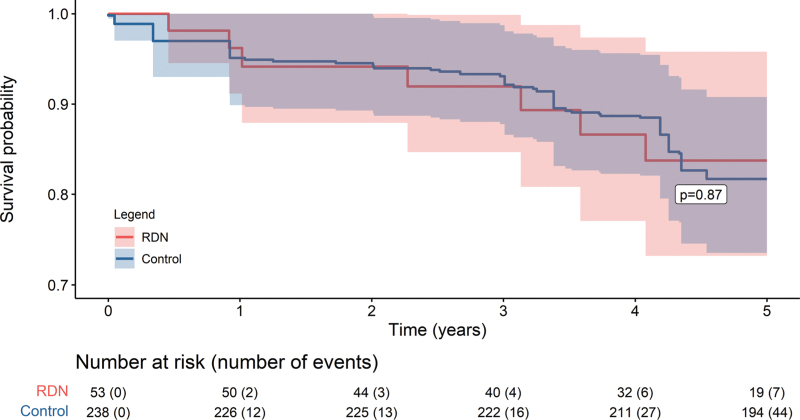
Occurrence of the primary safety outcome (composite endpoint) in the matched sample. CI, confidence interval; RDN, renal sympathetic denervation. Cox proportional hazards models with cluster-robust standard errors were performed to adjust for data clustering within matched pairs. Matching weights were applied in all models to adjust for variable ratio matching. The *P* value was derived from the robust score test.

The primary safety outcome occurred in 7 (13%) patients in the RDN group and 44 (18%) patients in the control group [hazard ratio 0.93 (95% CI 0.36–2.38); *P* = 0.87] (Fig. 2). The cumulative incidence of all-cause mortality at 5 years was significantly lower in the RDN group as compared to the control group [0 (0%) vs. 23 (9%) patients; *P* = 0.01]. No between-group differences were observed for the other secondary event outcomes (Table 3, Supplemental Figure 1).

**TABLE 3:** Occurrence of the individual components of the composite outcome and cardiovascular mortality

	RDN group (*n* = 53)	Control group (*n* = 238)	Hazard ratio^a^ (95% CI)	*P* value^a^
Composite outcome [*n* (%)]	7 (13)	44 (18)	0.93 (0.36–2.38)	0.87
Myocardial infarction [*n* (%)]	2 (4)	5 (2)	2.21 (0.75–6.52)	0.35
Coronary revascularization [*n* (%)]	3 (6)	13 (6)	1.05 (0.28–4.00)	0.94
Stroke [*n* (%)]	3 (6)	17 (7)	1.03 (0.26–4.12)	0.97
Renal failure^b^ [*n* (%)]	1 (2)	0 (0)	–	0.32
All-cause mortality [*n* (%)]	0 (0)	23 (9)	–	0.01
Cardiovascular mortality [*n* (%)]	0 (0)	9 (4)	–	0.09

CI, confidence interval; RDN, renal sympathetic denervation.

a Marginal hazard ratios were derived from Cox proportional hazards models, with cluster-robust standard errors to adjust for data clustering within matched pairs. Matching weights were applied in all models to adjust for variable ratio matching. *P* values were derived from robust score-tests.

^b^ Defined as estimated glomerular filtration rate ≤15 ml/min/1.73 m^2^ or requirement for dialysis.

For renal function (eGFR), similar reductions were observed at 5 years in the RDN group [−6.4 (95% CI −13.5 to 0.8) ml/min/1.73 m^2^; *P* = 0.08] and the control group [−6.3 (95% CI −8.9 to −3.7) ml/min/1.73 m^2^; *P* < 0.001], thereby not resulting in a significant between-group difference [0.0 (95% CI −7.9 to 7.9) ml/min/1.73 m^2^; *P* = 1.00].

### Efficacy outcomes

**FIGURE 3:**
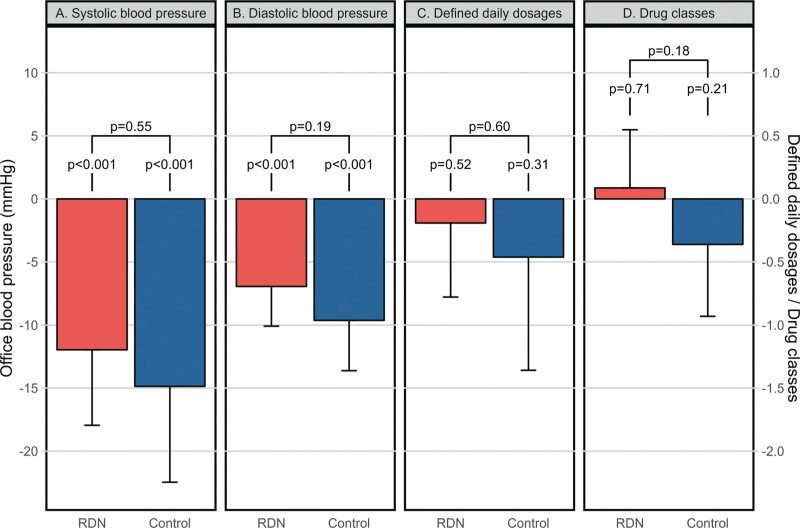
Changes in blood pressure and prescribed antihypertensive drugs between baseline and 5 years in the matched sample. RDN, renal sympathetic denervation.

Between baseline and 5 years, a decrease in SBP was observed both in the RDN group [−12 (95% CI −18.0 to −6.0) mmHg; *P* < 0.001] and in the control group [−14.9 (95% CI −22.5 to −7.3) mmHg; *P* < 0.001]. No significant between-group difference was observed [2.9 (95% CI −6.6 to 12.4) mmHg; *P* = 0.55] (Fig. 3a). For DBP, reductions were observed in the RDN group [−6.9 (95% CI: −10.1 to −3.8) mmHg; *P* < 0.001] and the control group [−9.6 (95% CI −13.6 to −5.6) mmHg; *P* < 0.001], while no significant between-group difference was observed [2.7 (95% CI −1.3 to 6.7) mmHg; *P* = 0.19] (Fig. 3b). At 5 years, BP control was achieved less frequently in the RDN group as compared to the control group [0 (0%) vs. 23 (23%) patients; *P* < 0.001].

The number of DDDs of antihypertensive drugs remained stable both in the RDN group [−0.2 (95% CI −0.8 to 0.4); *P* = 0.52] and the control group [−0.5 (95% CI −1.4 to 0.4); *P* = 0.31], and no significant between-group difference was observed [0.3 (95% CI −0.7 to 1.3); *P* = 0.60] between baseline and 5 years (Fig. 3c). Similar findings were observed for the number of antihypertensive drug classes (Fig. 3d).

### Sensitivity analyses

The source population included all eligible patients, irrespective of whether or not they could be propensity-matched, from both the RDN group (*n* = 82) and the control group (*n* = 1311). Within this population, the primary safety outcome occurred in 17 (21%) patients in the RDN group and 104 (8%) patients in the control group between baseline and 5 years (Supplemental Figure 2–3). Renal function (eGFR) declined over 5 years in both the RDN group [−8 (95% CI −11 to −5.1) ml/min/1.73−m^2^; *P* < 0.001] and the control group [−6.6 (95% CI −7.2 to −5.9) ml/min/1.73−m^2^; *P* < 0.001]. Significant office SBP reductions were observed in both the RDN group [−13.3 (95% CI −18.7 to −8.0) mmHg; *P* < 0.001] and the control group [−5.4 (95% CI −6.6 to −4.2) mmHg; *P* < 0.001] at 5 years. The number of DDDs remained stable in the RDN group [−0.3 (95% CI −0.7 to 0.1); *P* = 0.19], while this number increased in the control group [0.4 (95% CI 0.4–0.5); *P* < 0.001]. Similar findings were observed for the number of drug classes (Supplemental Figure 4). At 5 years, office BP control was achieved in 1 (2%) patient in the RDN group and 345 (35%) patients in the control group.

## DISCUSSION

To the best of our knowledge, this study was the first to compare long-term clinical event and BP outcomes between patients treated with RDN and population-sampled controls. Within our study, we observed no significant difference in cardiovascular risk between both groups. In parallel, we observed a sustained BP-lowering effect 5 years following RDN, which was not significantly greater than the BP changes observed in the control group. Antihypertensive drug burden remained stable in both groups over time.

The current work emphasizes the substantial risk associated with uncontrolled hypertension. We demonstrated a 5-year incidence of 13–18% for major cardiovascular and renal adverse events in our study population consisting of patients with uncontrolled hypertension despite a median of 2.8 prescribed DDDs of antihypertensive drugs. This event rate was almost two times as high as the 5-year event rate of 8% in an overall hypertensive population (as observed in the control group in the sensitivity analysis; Supplemental Figure 2). These findings demonstrate the unmet need for novel treatment modalities for patients in whom BP remains uncontrolled despite the use of a substantial number of antihypertensive drugs. The latter warrants more research on the effectiveness of new pharmacological agents, therapy adherence interventions and interventional therapies for hypertension.

Within the current study, RDN did not reduce the risk of the composite safety outcome as compared to matched population-based controls. However, a significant protective effect of RDN was observed for all-cause mortality, a finding that was driven by higher numbers of both cardiovascular and noncardiovascular mortality in the control arm. Given the low absolute number of events, a false-positive result cannot be ruled out. It could, however, be hypothesized that RDN could still have improved 24 h ambulatory BP (which was not routinely measured in all patients in the current study population) to a greater extent, which is in turn closely related to all-cause mortality [25]. With respect to treatment safety, renal function did not differ between the RDN group and the control group at follow-up, neither did the incidence of renal failure. Overall, our findings support the safety of RDN up until at least five years.

Within the RDN group, the 12 mmHg reduction in office SBP could be explained by a reduction in sympathetic nerve activity, with BP reductions generally comparable to previously reported effects on the long-term following RDN [15–19,26]. In parallel, an unexplained BP reduction was observed in the control group, which was not in line with the generally accepted concept of increasing BP with rising age [27,28]. Of note, as of to date, there is a lack of dedicated long-term longitudinal registry studies reporting on the evolution of BP levels and antihypertensive drug prescriptions over time. Within the current study, a regression-to-the-mean phenomenon could partly explain the unexpected decrease in BP, as office BP served both as an inclusion criterion and as a study outcome. The magnitude of this effect was likely larger in the control group due to differences in patient selection. More specifically, preprocedural 24 h ambulatory BP measurements were performed in all RDN patients to confirm uncontrolled hypertension (i.e. the indication for their procedure), whereas no ambulatory BP measurements were performed in control patients. The use of office BP over ambulatory BP is known to increase the risk of regression-to-the-mean [29]. Consequently, we cannot rule out a more severe regression-to-the-mean bias in the control group, resulting in an overestimation of the BP reduction within the control group and thereby leading to an underestimation of the between-group BP difference.

Antihypertensive drug burden over time was assessed using the number of DDDs to allow for comparison of the drug regimen between patients with different drug classes, types or dosages. Following propensity score matching, the number of DDDs (2.9 vs. 2.8) and the number of classes of antihypertensive drugs (2.5 vs. 2.5) at baseline were similar for the RDN group and the control group. Yet, calcium channel blockers and alpha blockers were more common in the RDN group, while mineralocorticoid receptor antagonists and beta blockers were less common. Renin–angiotensin–aldosterone pathway inhibitors were prescribed to a similar extent in both groups. Except for thiazides, which were prescribed equally among both groups in the current study, different antihypertensive drug classes comprised similar cardiovascular protective effects previously [30]. Nevertheless, we cannot rule out any (differential) effect of nonadherence to antihypertensive drugs, as this is common (46%) among patients with suspected refractory hypertension [31]. Until to date, there is no consensus on the assessment of nonadherence to antihypertensive drugs, and no interventions targeting adherence have demonstrated to improve BP or cardiovascular risk [32]. Overall, this precludes any strong conclusions on the effect of concomitant antihypertensive drugs on cardiovascular and renal outcomes in our study.

Within our study, all outcomes were also analyzed in the source population in the context of sensitivity analyses, in order to provide more insights in the data underlying the propensity-matched cohort. With regard to safety outcomes, the clinical event rate in the RDN group was numerically higher as compared to the control group, which could be explained by a more favorable cardiovascular risk profile in the control group (as displayed in Supplemental Table 2). The event data from the control group of the source population could serve as a useful metric for sample size calculation in future randomized studies on RDN focusing on clinical event rates. With regard to BP outcomes, a reduction of −13.3 mmHg in office SBP was observed at 5 years in the RDN group of the source population, which could not be explained by an increase in antihypertensive drug burden. These observations are in line with current literature on the long-term efficacy of the procedure [15–19]. Within the control group of the source population, in which office SBP and the number of antihypertensive drug DDDs at baseline were numerically lower (in comparison to the RDN group), a numerically smaller change in office SBP of −5.4 mmHg was observed, in parallel to an increase in antihypertensive drug burden. Overall, within the source population, BP reductions unaccompanied by an uptitration of antihypertensive drugs were observed in the RDN group but not in the control group. This could be explained by a reduction in sympathetic nerve activity following RDN.

### Limitations

This study has several limitations. First, this study was not statistically powered to detect any predetermined difference in clinical event outcomes between both groups, driven by a lack of dedicated registries on long-term clinical follow-up of hypertensive patients. Consequently, our study could have been underpowered for the clinical event outcomes. Second, propensity score matching was adopted to account for confounding bias. Nevertheless, any unmeasured confounding based on unobserved variables cannot be ruled out. Third, this study lacked 24 h ambulatory BP measurements in patients in the control group. As discussed above, this could have induced bias because of less severe hypertension in the control group (in contrast to the RDN group). Furthermore, the absence of annual ambulatory BP monitoring in the control group could have differentially affected the outcomes of this study. Fourth, no figures were available on office BP and antihypertensive drug outcomes in between baseline and 5 years, thereby precluding any statements on consistency of the observed effects. Finally, no therapy adherence measurements were performed in the current study. This precludes any statements about differences in therapy adherence at baseline and follow-up, as well as on the effect of adherence on BP over time.

### Recommendations for future research

Within the field of RDN, virtually all long-term follow-up studies lacked a control-arm, precluding any causal statements about the effect of the therapy on cardiovascular and renal adverse clinical outcomes [15–19]. The latter calls for adequately powered randomized clinical outcome trials. As compared to the current propensity-matched design, a randomized trial would resolve the risks of both confounding bias and selection bias (provided that follow-up will be complete in the absence of cross-overs to RDN).

As a more feasible alternative to large-scale randomized trials, the current study design of propensity score matching could be repeated. To improve the comparability of the controls to patients undergoing RDN at baseline, future longitudinal registry studies should be performed in high-risk hypertensive patients. These studies should focus on patients who receive routine medical therapy, including data collection on the change over time in ambulatory and office BP, prescribed drug regimen, therapy adherence and hypertension-mediated organ damage. These data could provide valuable new insights when embedded as a control group in studies on novel pharmacological (e.g. spironolactone) or interventional (e.g. RDN) antihypertensive treatment modalities.

## CONCLUSION

Patients with uncontrolled hypertension undergoing RDN did not have a significantly lower risk for future cardiovascular or renal adverse events as compared to a propensity-matched real-world cohort undergoing conventional follow-up, albeit a potential beneficial effect of RDN on all-cause mortality was noted. At 5 years, patients treated with RDN experienced a significant BP reduction, which was, however, not greater as compared to the BP reduction in control patients from a population-based study. Adequately powered randomized controlled trials are needed to confirm the potential benefit of RDN in improving long-term outcomes.

## ACKNOWLEDGEMENTS

We thank Dr Layal Chaker for her help in providing and interpreting the renal function data in the Rotterdam Study.

Ethics approval: this study was performed in line with the principles of the Declaration of Helsinki. Informed consent was obtained from all individual participants included in the study.

### Conflicts of interest

J.D. received institutional grant/research support from Abbott Vascular, Boston Scientific, ACIST Medical, Medtronic, Pie Medical, and ReCor medical, and consultancy and speaker fees from Abbott Vascular, Abiomed, ACIST medical, Boston Scientific, Cardialysis BV, CardiacBooster, Kaminari Medical, ReCor Medical, PulseCath, Pie Medical, Sanofi, Siemens Healthcare and Medtronic. All other authors declare no relevant interests to disclose.

## Supplementary Material

Supplemental Digital Content
