# Supplementary material for: Long-term safety and efficacy of renal sympathetic denervation in comparison to a population-based cohort: a propensity-matching approach
Source: J Hypertens. 2025 Aug 11;43(11):1832–40. doi: 10.1097/HJH.0000000000004117 (PMC12517731; doi:10.1097/HJH.0000000000004117)
Supplement: Supplemental Digital Content [file jhype-43-1832-s001.pdf]

Supplementary Material

Supplemental Table 1. Baseline characteristics in the cohort matched for efficacy outcomes

	**RDN group (n=23)**	**Control group (n=100)**	**P-value[1]**
Age (years), median [25th-75th percentile]	60.1 [54.6-65.6]	60.2 [56.7-67.0]	0.45
Female sex, n (%)	13 (57)	50 (50)	0.64
Body mass index (kg/m^2), median [25th-75th percentile]	24.5 [25.2-30.2]	28.7 [25.8-31.7]	0.25
Estimated glomerular filtration rate (ml/min/1.73m^2), median [25th-75th percentile]	67.0 [59.8-85.5]	75.0 [66.2-89.7]	0.70
**Cardiovascular risk factors**			
Hypertension, n (%)	23 (100)	100 (100)	1.00
Dyslipidemia, n (%)	19 (83)	92 (92)	0.29
Current smoking, n (%)	7 (30)	14 (14)	0.35
Former smoking, n (%)	3 (13)	55 (55)	
Diabetes mellitus, n (%)	4 (17)	24 (24)	0.48
**History of cardiovascular disease**			
Any cardiovascular disease, n (%)	4 (17)	26 (26)	0.47
Myocardial infarction, n (%)	1 (4)	5 (5)	0.95
Coronary revascularization, n (%)	3 (13)	13 (13)	0.98
Stroke, n (%)	1 (4.3)	3 (3)	0.67
Atrial fibrillation, n (%)	0 (0)	11 (11)	<0.001
**Office blood pressure**			
Systolic blood pressure (mmHg), mean ±SD	169.8 ±19.2	166.1 ±20.5	0.34
Diastolic blood pressure (mmHg), mean ±SD	97.7 ±12.8	95.2 ±10.4	0.40

**Antihypertensive drug regimen - summary measures**			
Number of defined daily dosages, median [25th-75th percentile]	3.3 [1.1-4.7]	2.8 [1.5-4.0]	0.70
Number of classes, median [25th-75th percentile]	2.5 [1.4-2.8]	2.5 [1.3-2.8]	0.39
**Antihypertensive drug regimen - drug classes**			
Thiazide diuretics, n (%)	15 (65)	62 (62)	0.71
Calcium channel blockers, n (%)	14 (61)	30 (30)	0.01
Angiotensin-converting enzyme inhibitors, n (%)	4 (17)	32 (32)	0.16
Angiotensin receptor blockers, n (%)	12 (52)	34 (34)	0.18
Mineralocorticoid receptor antagonist, n (%)	1 (4)	20 (20)	0.06
Alpha blockers, n (%)	1 (4)	3 (3)	0.78
Beta blockers, n (%)	8 (35)	66 (66)	0.001
Direct renin inhibitor, n (%)	0 (0)	0 (0)	1.00
Loop diuretics, n (%)	0 (0)	3 (3)	<0.001
**Procedural characteristics**			
Procedural duration (min), median [25th-75th percentile]	59.5 [52.3-68.0]	-	-
Contrast volume (ml), median [25th-75th percentile]	75.0 [60.8-98.8]	-	-
**RDN Device modality**			
Paradise (US), n (%)	9 (39)	-	-
Symplicity Spyral (RF), n (%)	8 (35)	-	-
Symplicity Flex (RF), n (%)	2 (9)	-	-
EnligHTN (RF), n (%)	2 (9)	-	-
Vessix (RF), n (%)	2 (9)	-	-
OneShot (RF), n (%)	0 (0)	-	-

Number of bilateral emissions			
Paradise (US), median [25th-75th percentile]	5.5 [4.1-5.6]	-	-
Symplicity Spyral (RF), median [25th-75th percentile]	18.0 [11.0-20.5]	-	-
Symplicity Flex (RF), median [25th-75th percentile]	7.0 [7.0-9.0]	-	-
EnligHTN (RF), median [25th-75th percentile]	8.0 [8.0-11.5]	-	-
Vessix (RF), median [25th-75th percentile]	4.0 [4.0-10.0]	-	-
OneShot (RF), median [25th-75th percentile]	N/A	-	-

RDN, Renal Sympathetic Denervation. RF, Radiofrequency. SD, Standard Deviation. US, Ultrasound.

[1] Adjusted for data clustering (within matched pairs) and matching weights (due to variable ratio matching).

Supplemental Table 2. Baseline characteristics in the unmatched cohort

	**RDN group (n=82)**	**Control group (n= 1,311)**
Age (years), median [25th-75th percentile]	63.0 [56.6-70.3]	57.8 [53.3-60.9]
Female sex, n (%)	38 (46)	676 (52)
Body mass index (kg/m^2), median [25th-75th percentile]	28.9 [26.1-32.6]	28.1 [25.6-31.1]
Estimated glomerular filtration rate ($ml/min/1.73m^2$), median [25th-75th percentile]	72.5 [62.0-85.0]	86.0 [76.0-95.5]
**Cardiovascular risk factors**		
Hypertension, n (%)	82 (100)	1,311 (100)
Dyslipidemia, n (%)	77 (94)	1,106 (84)
Current smoking, n (%)	17 (21)	324 (25)
Former smoking, n (%)	15 (18)	581 (44)
Diabetes mellitus, n (%)	18 (22)	174 (13)
**History of cardiovascular disease**		
Any cardiovascular disease, n (%)	34 (42)	109 (8)
Myocardial infarction, n (%)	12 (15)	29 (2)
Coronary revascularization, n (%)	23 (28)	31 (2)
Stroke, n (%)	9 (11)	37 (3)
Atrial fibrillation, n (%)	4 (5)	38 (3)
**Office blood pressure**		
Systolic blood pressure (mmHg), mean ±SD	171.9 ±18.8	151.0 ±14.4
Diastolic blood pressure (mmHg), mean ±SD	96.2 ±14.5	92.2 ±9.1
**Antihypertensive drug regimen - summary measures**		
Number of defined daily dosages, median [25th-75th percentile]	4.5 [2.5-6.0]	0.0 [0.0-1.0]
Number of classes, median [25th-75th percentile]	3.0 [2.3-4.0]	0.0 [0.0-1.0]

**Antihypertensive drug regimen - drug classes**		
Thiazide diuretics, n (%)	55 (67)	184 (14)
Calcium channel blockers, n (%)	51 (62)	79 (6)
Angiotensin-converting enzyme inhibitors, n (%)	15 (18)	119 (9)
Angiotensin receptor blockers, n (%)	49 (60)	126 (10)
Mineralocorticoid receptor antagonist, n (%)	12 (15)	35 (3)
Alpha blockers, n (%)	21 (26)	4 (0)
Beta blockers, n (%)	46 (56)	219 (17)
Direct renin inhibitor, n (%)	1 (1)	0 (0)
Loop diuretics, n (%)	3 (4)	16 (1)
**RDN procedural characteristics**		
Procedural duration (min), median [25th-75th percentile]	63.0 [53.0-75.0]	N/A
Contrast volume (ml), median [25th-75th percentile]	81.0 [62.5-127.5]	N/A
**RDN Device modality**		
Paradise (US), n (%)	27 (33)	N/A
Symplicity Spyral (RF), n (%)	21 (26)	N/A
Symplicity Flex (RF), n (%)	10 (12)	N/A
EnligHTN (RF), n (%)	18 (22)	N/A
Vessix (RF), n (%)	4 (5)	N/A
OneShot (RF), n (%)	2 (2)	N/A
**Number of bilateral emissions**		
Paradise (US), median [25th-75th percentile]	6 [5-6]	N/A
Symplicity Spyral (RF), median [25th-75th percentile]	23 [19-26]	N/A
Symplicity Flex (RF), median [25th-75th percentile]	10 [9-11]	N/A
EnligHTN (RF), median [25th-75th percentile]	16 [15-20]	N/A
Vessix (RF), median [25th-75th percentile]	8 [4-13]	N/A
OneShot (RF), median [25th-75th percentile]	2 [2-2]	N/A

RDN, Renal Sympathetic Denervation. RF, Radiofrequency. SD, Standard Deviation. US, Ultrasound.

Supplemental Table 3. Reasons for non-eligibility of RDN and control patients

	**RDN group (n=87)**	**Control group (n=3,428)**
Absence of history of hypertension , n (%)	0 (0.0)	1,733 (50.7)
Office blood pressure < 140/90 mmHg, n (%)	5 (5.7)[1]	2,114 (61.7)
Estimated glomerular filtration rate <30 ml/min/1.73m^2, n (%)	0 (0.0)	5 (0.1)

RDN, Renal Sympathetic Denervation.

[1] A total of five patients with symptomatic atrial fibrillation underwent RDN for with the primary aim to reduce their atrial fibrillation burden. While on antihypertensive drug therapy, these patients had controlled office blood pressure (systolic blood pressure <140 mmHg and diastolic blood pressure <90 mmHg) at baseline, and therefore they were excluded from the current study.

Supplemental Figure 1. Occurrence of the individual components of the composite outcome and cardiovascular mortality in the matched sample

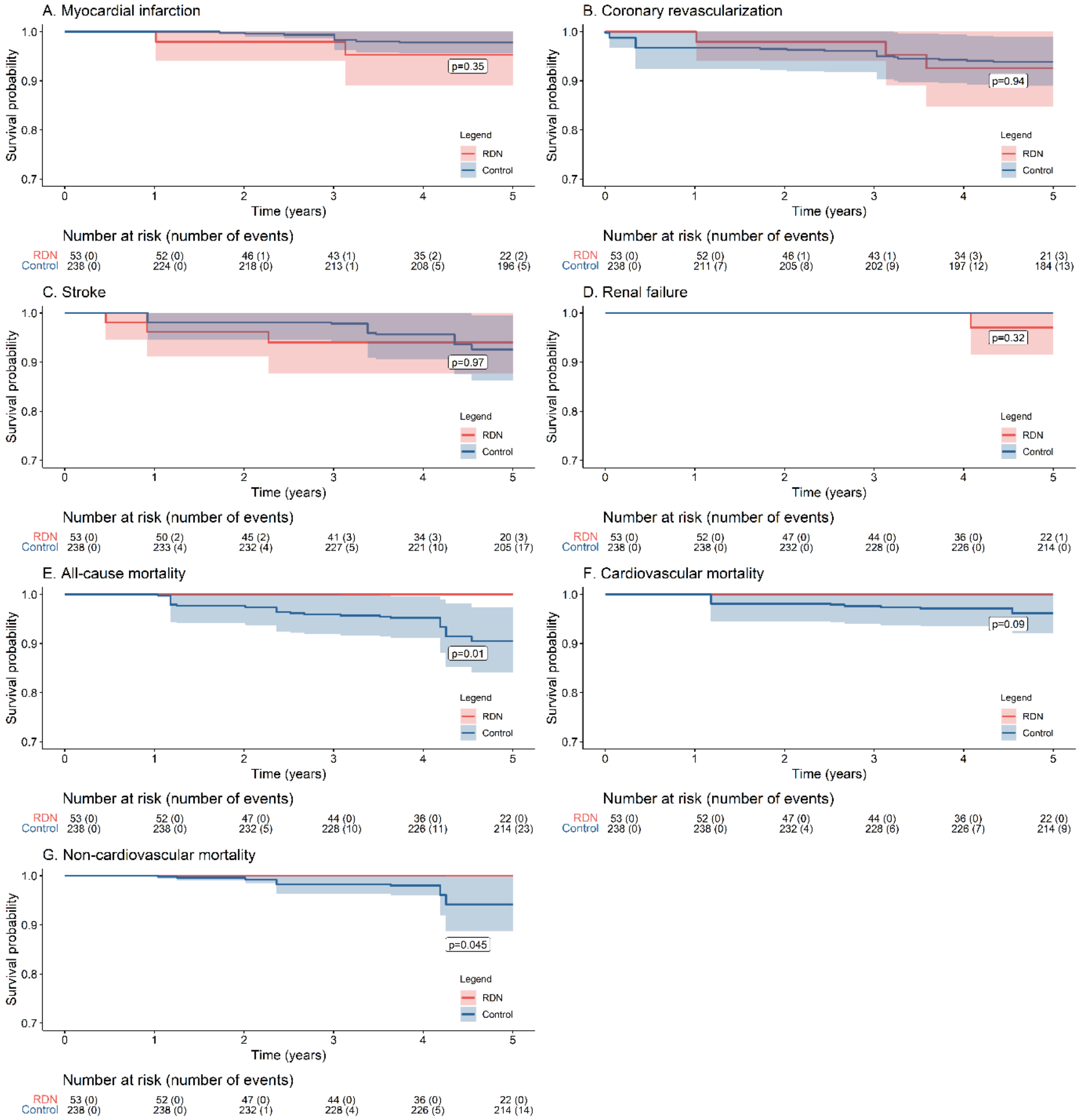

RDN, Renal Sympathetic Denervation.

Cox proportional hazards models with cluster-robust standard errors were performed to adjust for data clustering within matched pairs. Matching weights were applied in all models to adjust for variable ratio matching. The p-values were derived from the robust score-test.

Supplemental Figure 2. Occurrence of the primary safety outcome (composite endpoint) in the unmatched sample (sensitivity analyses)

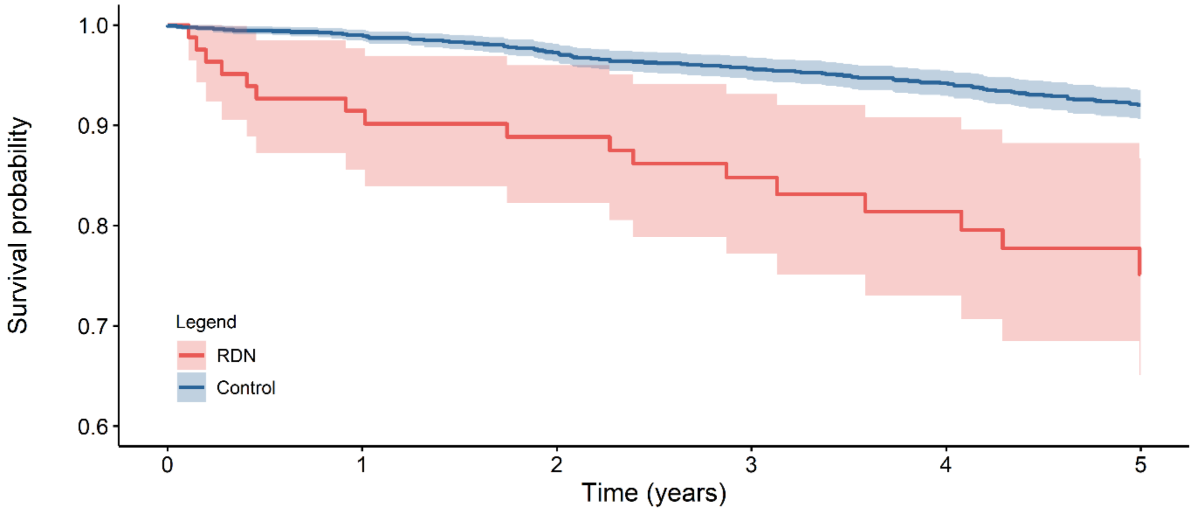

Number at risk (number of events)

	0	1	2	3	4	5
RDN	82 (0)	74 (7)	67 (9)	58 (12)	48 (14)	26 (17)
Control	1311 (1)	1297 (13)	1274 (36)	1251 (57)	1230 (76)	1200 (104)

CI, Confidence Interval. RDN, Renal Sympathetic Denervation.

Supplemental Figure 3. Occurrence of the individual components of the composite outcome and cardiovascular mortality in the unmatched sample

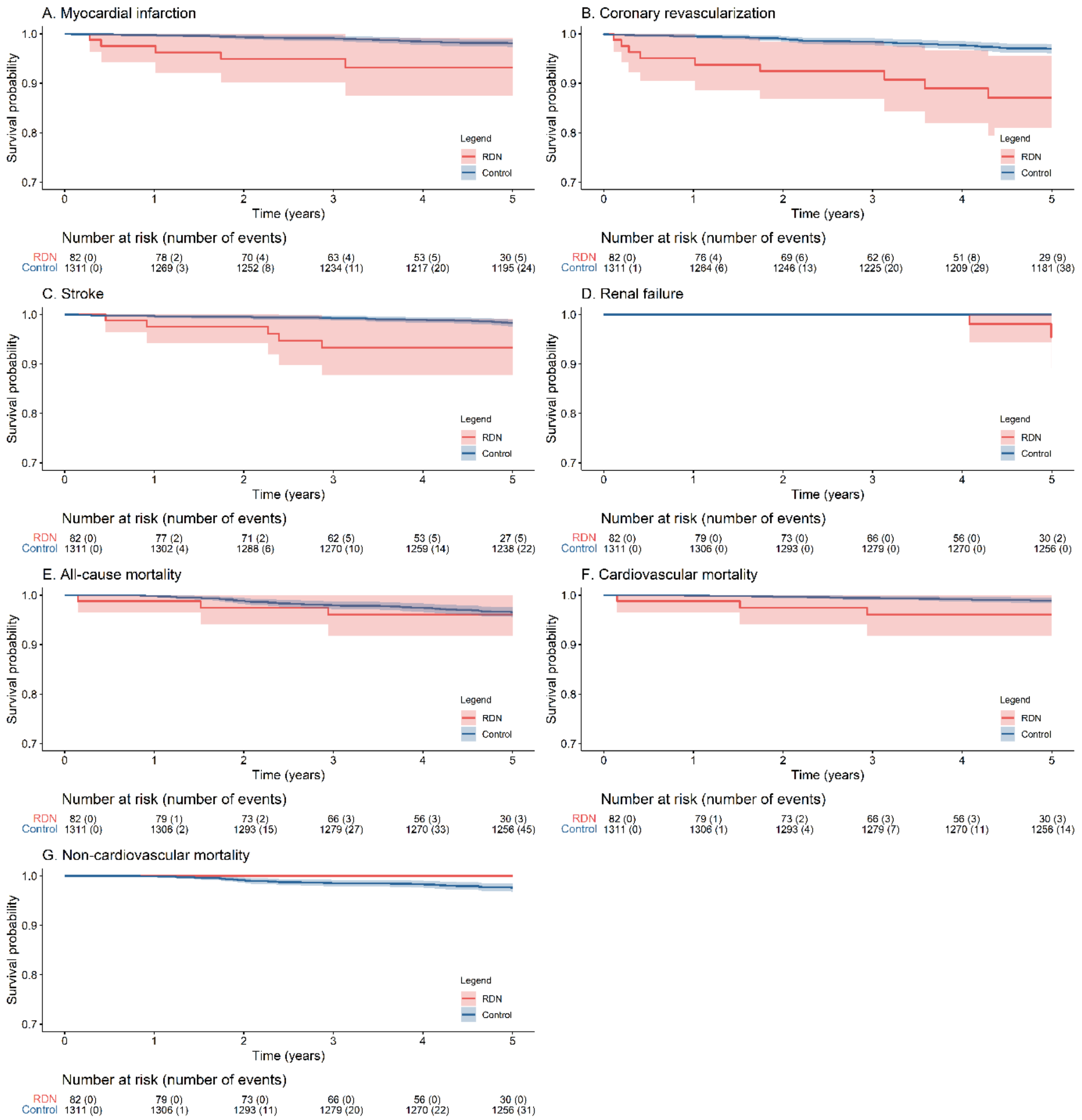

RDN, Renal Sympathetic Denervation.

Supplemental Figure 4. Changes in blood pressure and prescribed antihypertensive drugs between baseline and five years in the unmatched sample (sensitivity analyses)

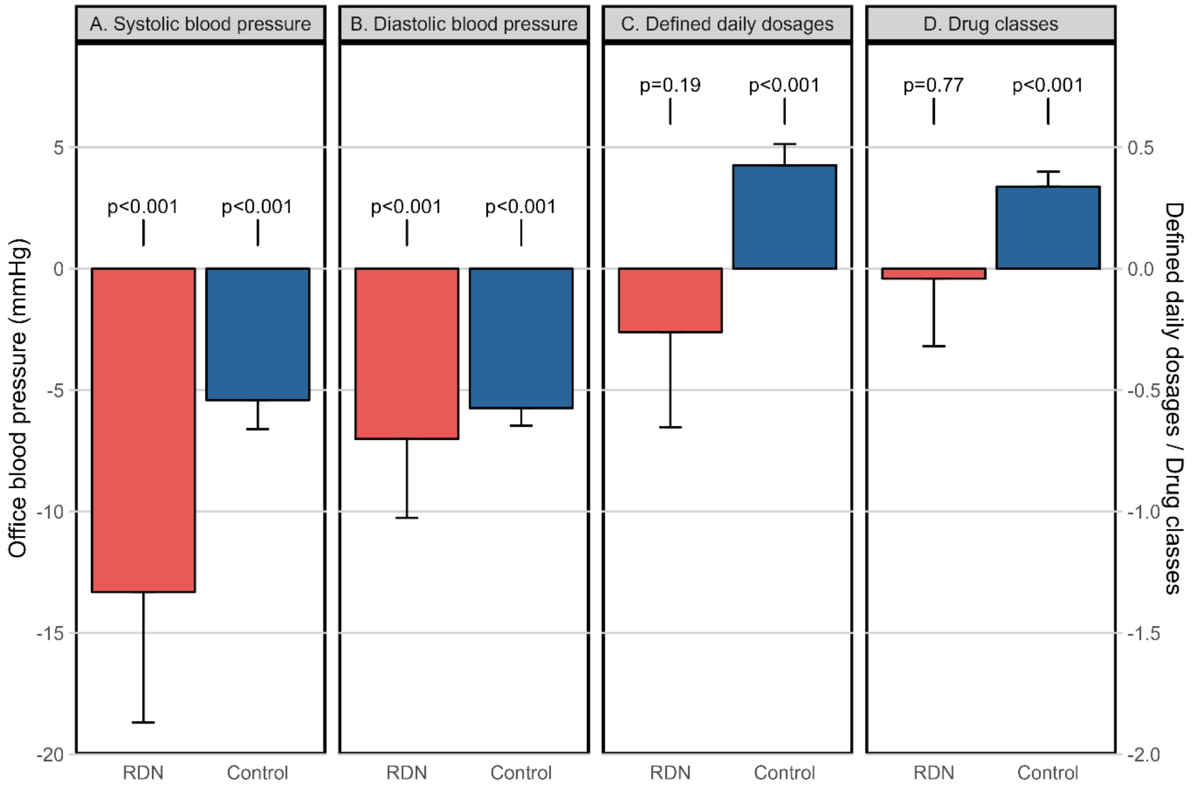

CI, Confidence Interval. RDN, Renal Sympathetic Denervation.

Supplemental Methods

Definitions:

Blood pressure control: office systolic blood pressure <140 mmHg and office diastolic blood pressure <90 mmHg.

Dyslipidemia: total cholesterol >5.2 mmol/L, low-density lipoprotein-cholesterol >3.4 mmol/L, high-density lipoprotein-cholesterol <0.9 mmol/L, triglycerides >1.7 mmol/L or the use of lipid-lowering medication.

Hypertension: office systolic blood pressure ≥140 mmHg or office diastolic blood pressure ≥90 mmHg.

Renal failure: defined as eGFR ≤15 ml/min/1.73m^2 or requirement for dialysis.

Calculations and procedures:

Office blood pressure: measurements were performed in seated position using manual or oscillometric cuff-based measurements at the level of the brachial artery. The measurement was performed twice and the average of both measurements was used for analyses.

Renal function: displayed using the estimated Glomerular Filtration Rate based on the CKD-EPI formula based on the serum creatinine [Levey AS, Stevens LA, Schmid CH, Zhang YL, Castro AF, 3rd, Feldman HI, et al. A new equation to estimate glomerular filtration rate. Ann Intern Med. 2009;150(9):604-12.].

Total number of defined daily dosages (DDD): the prescribed daily dose of each antihypertensive drug was divided by the predefined DDD by the World Health Organization [WHO Collaborating Centre for Drug Statistics Methodology. DDD—definition and general considerations: WHO Collaborating Centre for Drug Statistics Methodology; 2018. [https://www.whocc.no/ddd/definition_and_general_considera/]. The amount of DDDs prescribed within individual classes were then summed for all antihypertensive drug classes within each individual patient.

Source population:

RDN patients (Rotterdam Renal Denervation Registry): all RDN patients included between August 2012 and March 2022.

Control patients (Rotterdam Study): all participants from the RS-III cohort, included between February 2006 and November 2008.
